# Biosynthesis of planet friendly bioplastics using renewable carbon source

**DOI:** 10.1186/s40201-015-0165-3

**Published:** 2015-02-15

**Authors:** Roopesh Jain, Archana Tiwari

**Affiliations:** School of Biotechnology, Rajiv Gandhi Proudyogiki Vishwavidyalaya, Airport Bypass Road, Bhopal, Madhya Pradesh India

**Keywords:** Polyhydroxyalkanoates, Biosynthesis, Biodegradation, Cellulose acetate butyrate, Agricultural waste

## Abstract

Plastics are uniquely flexible materials that offer considerable benefits as a simple packing to complex engineering material. Traditional synthetic polymers (often called plastics), such as polypropylene and polyethylene have been derived from non-renewable petrochemicals and known to cause environmental concerns due to their non-biodegradable nature. The enormous use of petroleum-based plastic compounds emphasized a need for sustainable alternatives derived from renewable resources. Bioplastics have attracted widespread attention, as eco-friendly and eco-tolerable alternative. But they have got certain limitations as well, such as high cost of production and unsatisfactory mechanical properties. In this study we have found agriculture waste (AW) as low-cost and renewable substrate for the production of bioplastics in bacterial fermentation. Improvement in tensile properties of produced bioplastic film has also been documented upon blending with Cellulose Acetate Butyrate (CAB).

## Introduction

Every activity in modern life has been influenced by plastics and many depend entirely on plastic products due to their useful material properties and low production cost. Traditional synthetic polymers (often called plastics), such as polypropylene and polyethylene have been derived from non-renewable petrochemicals and known to cause environmental concerns due to their non-biodegradable nature [1]. The use of non-renewable fossil fuels for the production of plastic products has not only diminishing fossil fuel stockpiles but believed ‘eco-unfriendly’ too. However the need for polymers and the products they constitute has been reported ever increasing. The enormous use of petroleum-based plastic compounds emphasized a need for sustainable alternatives derived from renewable resources [2].

A special class of optically active biopolymers called Polyhydroxyalkanoates (PHAs) has been one of the most talked bioplastics and appeared as a potential candidate in replacing some petroleum based plastics because of their biodegradable nature and physical properties [3]. PHAs have showed some of the extraordinary similarities to the well-known synthetic polymers i.e. low-density polyethylene and polypropylene; in addition their disposal as bio-waste made them increasingly attractive in the pursuit of sustainable development of biodegradable plastics [4]. These PHAs comprised a class of polyesters of natural origin which has been accumulated by a variety of microorganisms intracellularly in the form of intracellular granules and stored in response to an environmental stress or nutrient limitation as a reserve of carbon, energy, and reducing power [5]. Under the conditions of carbon source depletion, PHAs have been reported to degrade by intracellular depolymerases and subsequently metabolized as a carbon and energy sources [6]. Poly-3-hydroxybutirate (PHB) and its copolymers with 3-hydroxyvalerate (3HV) known as poly(3-hydroxybutyrate-co-3-hydroxyvalerate) (PHBV), have been documented as the best known representatives of PHA family [7].

PHAs produced from standard carbon sources have got certain limitations such as high cost of production. Plant residues have been known as eco-friendly in nature and did not cause any ill-effects to the both flora and fauna. The best reported use of these agricultural wastes (AW) was feeding the cattle. Due to sufficient availability and non-commercial importance these natural residues have created attention for production of cost effective bioplastics [8].

In this study model strain *Cupriavidus necator* was used for the production of biopolymer. AW was studied as a cheap and renewable carbon source and compared with standard carbon source glucose in bacterial fermentation. Role of blending in produced bioplastics was also described.

## Materials and methods

### Growth media

Nutrient broth (NB) comprises distilled water, 5 g/L Peptic digest, 5 g/L Sodium chloride, 1 g/L Beef extract and 1/L Yeast extract.

Nutrient agar (NA) comprises distilled water, 5 g/L Peptic digest, 5 g/L Sodium chloride, 1 g/L Beef extract, 1 g/L Yeast extract and 15 g/L Agar.

Tryptone soya broth (TSB) comprises distilled water, 17 g/L Tryptone, 3 g/L Soy Peptone, 5 gl/L NaCl, 2.5 g/L K_2_HPO_4_ and 2.5 g/L D-glucose.

Tryptone soya agar (TSA) comprises distilled water, 15 g/L Tryptone, 5 g/L Soy Peptone, 5 g/L NaCl and 12 g/L Agar.

Basal salts medium (BSM) comprises distilled water, 1 g/L K_2_HPO_4_, 1 g/L KH_2_PO_4_, 1 g/L KNO_3_, 1 g/L (NH_4_)_2_SO_4_, 0.1 g/L MgSO_4_.7H_2_O, 0.1 g/L NaCl, 10 ml/L.

Trace element solution comprises 2 mg/L CaCl_2_, 2 mg/L CuSO_4_.5H_2_O, 2 mg/L MnSO_4_.5H_2_O, 2 mg/L ZnSO_4_.5H_2_O, 2 mg/L FeSO_4_, 2 mg/L (NH_4_)_6_Mo_7_O_24_.4H_2_O [9].

### Microorganism

The gram-negative bacterium *Cupriavidus necator* (former *Ralstonia eutropha*) was used as production culture and revived in nutrient broth. Cultures were tested for purity by Gram stain and observation of cell morphology under a microscope at 1000 × [9].

### Quick screening for PHB production

10 μl of 48 hr old culture of the isolate was transferred to an eppendorf tube containing 50 μl of acridine orange and incubated for 30 minutes at 30°C. After the incubation period, the culture was centrifuged at 4000 rpm, for 5 min. The pellet was collected and then resuspended in distilled water. A smear was prepared on a clean microscopic slide and observed in a fluorescent microscope at 460 nm [10].

### Microbial fermentation

TSB was inoculated from a single colony of *Cupriavidus necator* and incubated for approximately 24 hours at 30°C. Cultures were checked for purity by Gram staining and observed under a microscope at 1000 ×. BSM was used for batch fermentation [9]. 25ml TSB inoculums was added to 250 ml of BSM resulting in an inoculation ratio of 10% (v/v). AW was added to the fermentation medium as test carbon source while glucose as control at three different concentrations i.e., 10, 20 and 30 g/l. Batch fermentation was carried out using rotary incubator at 30°C and the growth of bacteria was checked intermittently after 24 hrs, 48 hrs and 72 hrs. All experiments were performed in triplicate. The biomass was separated by centrifugation.

### Recovery of PHB

The recovery of PHB from dried biomass was carried out by digestion method using sodium hypochlorite as described by Hahn *et al*. [4].

### Quantification of PHB

The Spectrophotometric method was used for quantification. Absorbance at 235 nm was measured against a sulphuric acid blank. Pure PHB was used to prepare the standard curve [11]. By referring to the standard curve, the quantity of produced PHB was determined.

### Blending of polymers and film fabrication

The blend films were prepared by conventional solvent casting technique [12]. Cellulose Acetate Butyrate (CAB) was used as blending agent in different ratios (PHB:CAB - 50:50, 60:40, 70:30, 80:20, 90:10 and control was 100% PHB).

### Testing of tensile strength and biodegradation

Tensile strength determines the breaking point of various items. Testing of PHB film and blends was performed using an instrument called “INSTRON”.

Laboratory testing is a common technique used to determine the susceptibility of compounds to biodegradation. In the lab test, solvent cast films of PHB and PHB blend (test samples), 100% biodegradable plastic (positive control) and synthetic plastic (negative control) of equal sizes were taken and weighed. All samples were added to separate container containing 100 g of garden soil and incubated for 6 months. Samples were periodically removed (once in 15 days), washed with distilled water and dried before analysis. The dried films were weighed for monitoring weight loss. All experiments were done in triplicate.

### Toxicity test

Toxicity studies were performed by 7 day plant seedling test (Cress test) [13].

## Results and discussion

**Figure 1:**
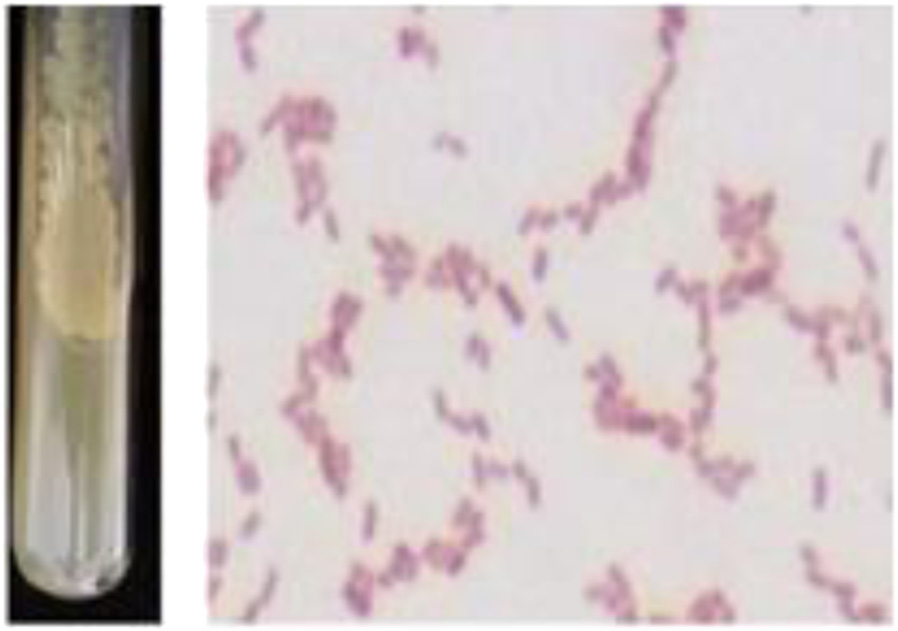
***Cupriavidus necator*** **colonies on slant and Gram staining of the revived culture.**

In this study standard carbon source glucose was compared with AW for PHB production in *Cupriavidus necator* fermentation. Nutrient broth was used for the revival of bacterial culture. Sub-culturing was carried out using TSA after regular intervals of 15 days to maintain the stock & purity [9]. Microscopic analysis of heat fixed cells was performed after Gram staining. *Cupriavidus necator* was characterized as gram negative, short rods shaped aerobic bacteria as shown in Figure 1. Rapid screening of bacteria for PHB production was carried out using acridine orange staining. Microbial colonies were able to incorporate the dye and appeared yellow-orange indicating PHB production [1].

Different concentration of glucose and AW were checked in the fermentation to determine maximum PHB accumulation in *Cupriavidus necator* cells. Both glucose and AW showed best results at 20 g/l in BSM after an incubation of 72 hours with maximum production at 48 hours. Data shown in Table 1 indicated that the maximum cell dry weight (CDW) and PHB concentration were 2.744 ± 0.005 gL^−1^ and 1.518 ± 0.026 gL^−1^ (55% of CWD) respectively with glucose; while 2.251 ± 0.012 gL^−1^ and 1.100 ± 0.125 gL^−1^ (49% of CWD) respectively with AW.

**Table 1:** **Comparison of CDW and PHB concentrations**

**Carbon source**	**Cell dry weight**	**PHB concentration**
	**(gL** ^**−1**^ **)**	**(gL** ^**−1**^ **)**
Glucose	2.744 ± 0.005	1.518 ± 0.026
AW	2.251 ± 0.012	1.100 ± 0.125

Values are written as the mean (gL^−1^) ± standard deviation (SD) of three replications.

Fermentation based commercial polymer production occurs in aerobic processes; therefore, only about 50% of the main carbon source has been used for production of biopolymers. A second cost factor in normal production processes for PHAs is the costs for complex nitrogen sources. Both cheap carbon sources and cheap nitrogen sources are available from agricultural waste and surplus materials [14]. Results encourages that the AW can act as a competent substrate to glucose for PHB production at 2% concentration.

PHB was recovered through sodium hypochlorite digestion method and biopolymer film was prepared by dissolving the extracted PHB in chloroform. Film was found to be brittle and broke easily. To overcome these weaknesses blending with CAB was carried out. CAB was reported as a FDA approved biodegradable and transparent thermoplastic similar to cellulose acetate but tougher with lower moisture absorption and better weathering resistance [15]. A varying range of blending agent CAB was used and compared with 100% standard PHB film.

**Figure 2:**
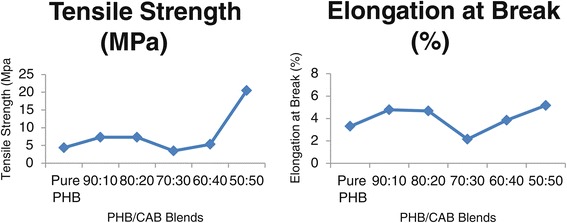
**Tensile properties of PHB/CAB blends.**

The main interest of plastic film preparation was to increase the amorphous content. The PHB was blended with CAB in ratios 50:50, 60:40, 70:30, 80:20, 90:10, while control was pure PHB. The tensile strength and elongation at break (Table 2) were tested to know the effect of blending against pure PHB film. Tensile strength value of Pure PHB was reported 4.363 ± 0.002 MPa, which was in the range of commercial PHB reported earlier [15-17]. Tensile strength of PHB/CAB (50:50 to 90:10) blends was varied from 7.303 to 20.397 MPa with best at 20.397 MPa for 50:50, whereas elongation at break varied from 2.149 to 5.158% with 5.158% as best for 50:50 blend (Figure 2). It was evident from results that blending had improved tensile propertied due to intermolecular bonding and increase in amorphous content [18,19].

**Table 2:** **Tensile properties of PHB/CAB blends**

**Sample**	**Tensile strength (MPa)**	**Elongation at break (%)**
Pure PHB	4.363 ± 0.002	3.298 ± 0.002
90:10	7.303 ± 0.014	4.773 ± 0.008
80:20	7.316 ± 0.002	4.671 ± 0.082
70:30	3.468 ± 0.002	2.149 ± 0.104
60:40	5.319 ± 0.010	3.839 ± 0.285
50:50	20.397 ± 0.368	5.158 ± 0.176

**Figure 3:**
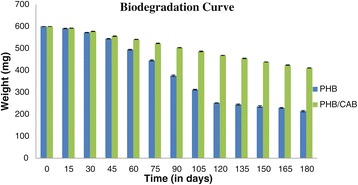
**Biodegradation Curve.**

The overall degradation mechanism involves abiotic factors followed by enzymatic degradation through PHB depolymerases and other degradation enzymes. These enzymes disrupt the inner molecular structure of biopolymer through microbial attack or microbial colonization. Therefore, the biodegradation efficiently begins from surface and gradually wrap the inner molecular conformation. Biodegradability analysis was carried out in lab tests for six months. PHB/CAB blend at ration 50:50 showed best physico-mechanical properties therefore was compared with pure PHB to find out biodegradation pattern. The readings for weight loss for blend and pure PHB were recorded after every 15 days and the same have been illustrated in biodegradation curve (Figure 3). PHB has showed 64.3% degradation in 6 months, whereas PHB/CAB blending exhibited only 31.5%. The miscibility of PHB and CAB was found very high that imparted highly amorphous nature to the blend, but at same times it made the polymer surface hydrophobic leading to a slow hydrolysis environment.

The toxicity study was performed by Cress Test, according to standard OECD guidelines. The cress, a dicotyledonous plant was chosen because of its fast germination rate and high sensitivity to the toxic chemicals [20]. At the end of the test, all emerged plants were found healthy with more than 90% germination rate for all treatment groups. Results of cress test demonstrated that the final degradation product of bioplastic was not toxic to plants.

## Conclusion

As environmental concerns became more prominent, it encouraged bigger debate and greater research in the bioplastics sector expanding the range of cost effective biodegradable products. Bioplastics have now been taken seriously because of many documented benefits such as saving fossil resources, climate protection and creating jobs in future-oriented sectors. Since the bioplastics originated from a renewable source, they have been believed much more environmental friendly than ‘non-renewable’ conventional plastics.

The major drawbacks for PHAs production are their production cost, and lack of comparable properties to the extent of adequate commercialization. The development of cost effective bioplastics is required to be necessarily preceded by investigations on the production factors with particular reference to the nature of micro-organisms used in the fermentation and the raw materials such as carbon source. This study has emphasized use of AW as a cost-competitive carbon source. Blending with approved biodegradable materials such as CAB has also attracted as an approach to manufacture commercially compatible bioplastics.
